# Prevalence and variability of HIV/AIDS-associated neurocognitive impairments in Africa: a systematic review and meta-analysis

**DOI:** 10.1186/s12889-023-15935-x

**Published:** 2023-05-30

**Authors:** Birhanie Mekuriaw, Zelalem Belayneh, Worku Teshome, Yonas Akalu

**Affiliations:** 1 Department of Psychiatry, College of Health and Medical Science, Dilla University, Dilla, Ethiopia; 2 School of Nursing and Midwifery, Faculty of Health, University of Technology Sydney, Sydney, Australia; 3 School of Primary and Allied Health Care, Faculty of Medicine, Nursing and Health Sciences, Monash University, Melbourne, Australia; 4 Department of Internal Medicine, College of Medicine and Health Science, Bahir-Dar University, Bahir-Dar, Ethiopia; 5 Department of Physiology, School of Medicine, College of Medicine and Health Science, University of Gondar, Gondar, Ethiopia

**Keywords:** Neurocognitive impairment, Dementia, Cognitive functioning, ART, HIV/AIDS, Comorbid

## Abstract

**Background:**

HIV/AIDS-associated neurocognitive impairments negatively affect treatment adherence, viral load suppression, CD4 count, functionality, and the overall quality of life of people with seropositive status. However, huge variability is observed across primary studies regarding the prevalence and determinants of neurocognitive impairment in people with HIV/AIDS. This systematic review and meta-analysis sought to determine the pooled prevalence of neurocognitive impairment and identify factors contributing to variations in its estimate among people living with HIV/AIDS in Africa.

**Methods:**

A comprehensive literature search of scientific databases (Medline/PubMed, SCOPUS, Web of Science, PsycINFO, and EMBASE) was performed from inception onward. Google and Google Scholar were also searched for grey literature. Research articles available until July 15, 2022 were included. We used STATA-version 14 statistical software for analysis. A random effect model was executed to pool the reported prevalence of neurocognitive impairments. Subgroup analysis was done to show variations in the prevalence of neurocognitive impairments and factors that might contribute to these variations.

**Results:**

A literature search resulted in 8,047 articles. After the removal of duplications and thorough evaluation, a total of 49 studies were included in the meta-analysis. The prevalence of HIV/AIDS-associated neurocognitive impairments was highly variable across studies, ranging from 14% to 88%, yielding the pooled prevalence of HIV/AIDS-associated neurocognitive impairment to be 46.34% [95% CI (40.32, 52.36)] and I^2^ = 98.5% with a *P*-value of 0.001.

**Conclusions:**

A large proportion of people living with HIV/AIDS in Africa have HIV/AIDS-associated neurocognitive impairment. This illustrates the need to establish practical approaches to early identification and effective control of HIV/AIDS-associated neurocognitive impairments. However, there were variabilities in the reported prevalence of HIV/AIDS-associated neurocognitive impairments across studies. This further demonstrates the need to have consistent measurement approaches.

**Trial registration:**

PROSPERO 2022, “CRD42020166572”.

**Supplementary Information:**

The online version contains supplementary material available at 10.1186/s12889-023-15935-x.

## Introduction

Human immunodeficiency virus (HIV) is a global epidemic that affects more than one-third of the world’s population. It is a neurotropic virus that affects the sub-cortical brain structure and may cause mild to severe neurological and cognitive impairments [1, 2]. Cognitive impairments in patients due to HIV/AIDS, sometimes called HIV-associated neurocognitive disorder (HAND), is a collective term used to describe three levels of cognitive impairments including, asymptomatic neurocognitive impairment, mild neurocognitive disorder, and frank dementia [1, 3]. HAND can cause mild to profound neurological deficits in the speed of problem-solving, decision-making ability, abstract thinking, memory, attention, and overall cognitive abilities of people with HIV/AIDS [4].

Significant increases in life expectancy for people with HIV/AIDS have been recorded after widespread coverage and early initiation of antiretroviral therapy (ART) drugs, and HIV/AIDS has become a treatable chronic condition. However, the presence of comorbid neurocognitive impairments leads HIV/AIDS to remain as a public health burden [5, 6].

Neurocognitive impairments and ART have reciprocal relationships. HIV/AIDS-related conditions like ART treatment non-adherence, immune suppression, late initiation of anti-retroviral therapy, potential ART drug side effects, comorbid medical and mental conditions, poor social support level, and other HIV/AIDS-related opportunistic infections contribute to the incidence of HIV/AIDS-associated neurocognitive problems [7, 8]. The presence of comorbid neurocognitive impairments, on the other hand, causes poor ART adherence, lower immune suppression, the development of opportunistic infections, poor decision making about their lifestyle, a lower survival rate, a decrease in CD4 levels, and the development of comorbidity with other primarily mental or medical conditions [9–11]. This may cause initiation of substance use and over-involvement in risky sexual behaviors, thereby increasing the risk of further HIV transmission and negatively affecting the overall patient's treatment outcome [7, 12]. In the UNAIDS global report on the global AIDS epidemic, HIV/AIDS-associated neurocognitive impairment was one of the contributing factors for 75% of the world’s burden of AIDS-related deaths in Africa [13, 14].

The magnitude of HIV/AIDS-associated neurocognitive impairments and trends over time are unclear. For example, a study conducted in Kenya reported the prevalence of HIV/AIDS-associated neurocognitive impairments to be 88%, while a study from Malawi recorded 14% [15, 16]. Similarly, factors associated with HIV/AIDS-associated neurocognitive impairments are inconsistent between studies [17]. This makes it hard to establish intervention strategies and compare their impact on reducing the burden of HIV/AIDS-associated neurocognitive impairments without valid and reliable data [18]. This calls for the need to synthesize summarized evidence on the prevalence of HIV/AIDS-associated neurocognitive impairments in Africa. Evidence on the overall burden of neurocognitive impairments is vital and seems to be the primary step in establishing policies, strategies, and guidelines to prevent and manage neurocognitive impairments among people with HIV/AIDS [14]. Therefore, this systematic review and meta-analysis aimed to pool the prevalence of HIV/AIDS-associated neurocognitive impairments and identify factors potentially contributing to variations in their prevalence estimates.

### Objectives

This systematic review has two main objectives: 1) pooled prevalence of HIV/AIDS-associated neurocognitive impairments; and 2) factors contributing to variations in the prevalence estimates of HIV/AIDS-associated neurocognitive impairments.

## Methods

### Reporting and protocol registration

This systematic review and meta-analysis followed the Preferred Reporting Items for Systematic Reviews and Meta-analysis guideline (PRISMA-P) protocol (Supplementary file-1). The review protocol has been registered in the International Prospective Register of Systematic Reviews (PROSPERO) “CRD42020166572.”

### Outcome measurements

Data for the prevalence of neurocognitive impairment was extracted from the direct reports of primary studies or determined by dividing the number of people with neurocognitive impairments by the total number of participants and multiplying by 100. Some papers used different terminologies (neurocognitive disorder, dementia, HIV/AIDS-associated dementia, neurocognitive impairment, cognitive dysfunction, …) rather than using the statement "neurocognitive impairment", and we took the reported magnitude for the first objective. Variables like study design, publication years, geographical location of studies, screening tools, and ART status of patients were evaluated to determine if each variable contributed to the variations in the reported prevalence of HIV/AIDS-associated neurocognitive impairments.

### Search strategy

A systematic search of scientific databases (PubMed/Medline, SCOPUS, Web of Sciences, PsycINFO, and EMBASE) was conducted from inception onward to retrieve research articles. The database search was conducted using key terms and Medical Subject Heading (MeSH) following a revised PICO (Population, Intervention, context/concept and outcome) approach where "Population" stands for concepts related to people with HIV/AIDS, "Context/Concept" represents clinics with HIV/AIDS-related services, "Intervention" covers neurocognitive impairment related topics, and "Outcome" represents concepts for the magnitude, prevalence, proportion and incidence rate related themes (Supplementary file-2). Grey literature were also searched from Google and Google Scholar. In addition, reference lists of all included articles were also manually checked for the availability of eligible primary studies.

### Study selection and eligibility assessment

Two authors (BM and ZB), independently evaluated the eligibility of primary studies using the predefined eligibility assessment criteria in accordance with the Preferred Reporting Items for Systematic Reviews and Meta-Analyses (PRISMA) guideline [19]. First, all retrieved articles were entered into the End-Note citation manager, and duplicated articles were excluded. All the remaining papers were then, screened by reading their titles/abstracts. Studies considered relevant during title/abstract evaluations were eligible for further full-text screening.

### Inclusion criteria

We included research articles meeting the following criteria: Both published and unpublished research studies that have been written in English language or having English translations; primary studies having result reports of neurocognitive-related concepts either as primary or secondary outcomes; studies conducted in any country of Africa; participants of people with HIV/AIDS (on ART or not), and with age ranges of 16 and above. Studies with different designs (cross-sectional, survey, pre-post-tests, case–control, trials, RCT, experimental, and cohort studies) were considered eligible, but only observational studies were found and included in this review. There was no publication year restriction, and articles available online until July 15, 2022 were included.

### Exclusion criteria

A study was excluded if: a) it was a review, letter, magazine article, commentary, legal paper, ethics paper, newspaper article, case study, qualitative paper, poster abstract or dissertation, opinion, or conference heading; b) it was published in a non-English language or did not have English language translation; c) it was conducted with mixed populations and no separate data for people with HIV/AIDS; or d) it was conducted in non-African countries.

### Quality assessment

Two authors (ZB and BM), independently evaluated the overall qualities of all included primary articles using quality assessment criteria adapted from the Joanna Briggs Institute Meta-Analysis of Statistics Assessment and Review Instrument (JBIMAStARI) [20]. The tool has its own appraisal checklist for each design. This criterion was considered to evaluate the description of the study subject and setting, valid and reliable measurement of exposure, objective and standard criteria used to identify and handle confounders, outcome measurement, and appropriate statistical analysis. Studies were deemed low risk of bias when they scored a quality evaluation indicator of 50% or above. Inconsistencies between the two assessors were resolved through discussion.

### Data extraction

Data were extracted from original studies using a predefined spreadsheet prepared in Microsoft Excel. The data extraction template was piloted by randomly selecting 10% of the included studies, and all authors approved it before the actual data extraction. During data extraction, there were different columns, each standing for a single variable (first author’s name, publication year, country where the study was conducted, screening tool used, study design, sample size, response rate, and prevalence of neurocognitive impairment), and each row represented the data of one primary study.

### Statistical procedures

First, the extracted data were exported from Microsoft Excel to STATA Version 14.0 (software) for analysis. Then, the standard error of neurocognitive impairment was calculated for each original article using a binomial distribution formula. The heterogeneity of papers was checked using the I^2^ statistics test [21]. The result of these tests showed that the data for the prevalence of neurocognitive impairments were heterogeneous among the included studies (I^2^ = 98.5%, a P-value of 0.001). Accordingly, Der Simonian and Laird’s pooled effect of neurocognitive impairment was estimated using a random-effects meta-analysis. Potential small study effects (publication bias) of primary studies were also evaluated by visual assessment of the symmetrical distribution of the funnel plot and Egger’s test statistics at a 5% significant level [22, 23].

We computed a sub-group analysis for the prevalence of HIV/AIDS-associated neurocognitive impairments with factors that might reduce the heterogeneity of the data reported (geographical locations of studies conducted, study design, measurement tools, and participants’ ART status).

## Results

### Search results

**Fig. 1:**
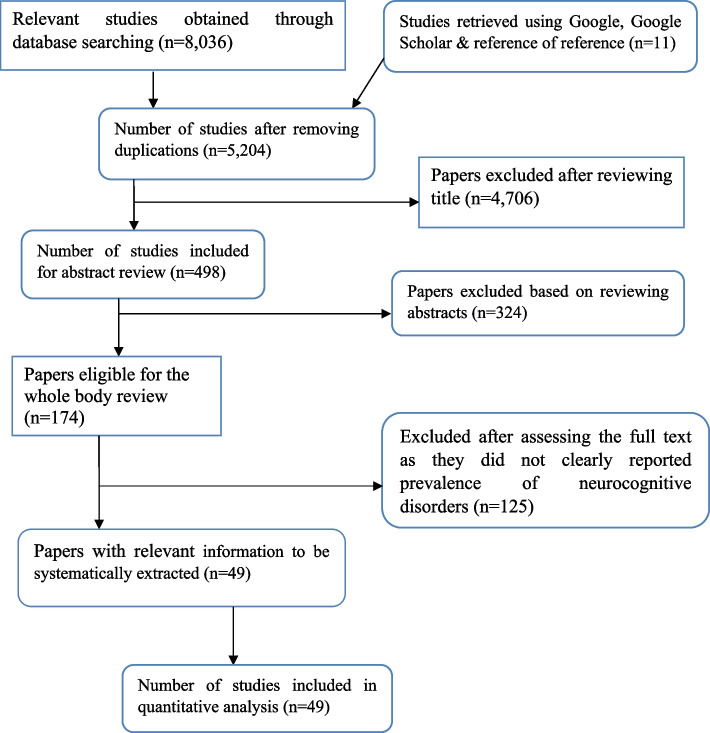
Diagramatic presentation of the articles selection process

The database and manual search of literature yielded a total of 8,047 articles. Of these articles, 2,843 articles were removed due to duplication and the other 5,030 were excluded after title/ abstract screening. The remaining 174 primary studies were considered for full text review and 125 studies were further excluded due to variations in study participants, outcome interests and study settings (not in Africa). Finally, 49 articles were found to be eligible and included in the systematic review and meta-analysis (Fig. 1).

### Original characteristics of included studies

A total of 15,029 study participants were included in the overall studies included in this meta-analysis (*n* = 49). More than half of the included studies (*n* = 32) used the International HIV Dementia Scale (IHDS) to measure the prevalence of neurocognitive impairments. The IHDS is a validated tool used to screen HAND among individuals for dichotomous outcomes (absence and presence) in different Sub-Saharan countries with good sensitivity and specificity [24–26]. The tool does not show the level/severity of neurocognitive impairments, and the clinical diagnosis of neurocognitive disorder has not been confirmed. However, most of the included studies used the IHDS with its limitations. A total of 30 studies were conducted among people on ART, while the number of studies conducted with ART-naive, and both ART and naive populations were 6 and 9, respectively. All papers have acceptable response rates (89% and above) and fulfilled the quality assessment score (Table 1).

**Table 1:** Original characteristics of studies included in the systematic review and meta-analysis of neurocognitive impairments in people living with HIV/AIDS in Africa, 2022 (*n *= 49).

First author’s name	Publication year	Country	Sample size	Response rate (%)	Measurement tools	Risk of bias	Prevalence	Study design	ART status of enrolled participants
Animut D. et al. [27]	2019	Ethiopia	697	98	IHDS	Low risk	67.1%	Cross-sectional	On ART
Belete et al. [28]	2017	Ethiopia	254	92.1	IHDS	Low risk	33.3%	Cross-sectional	On ART
Nakku et al. [24]	2013	Uganda	680	90.9	IHDS	Low risk	64.4%	Cross-sectional	On ART
Mossie B et al. [29]	2014	Ethiopia	423	100	IHDS	Low risk	24.8%	Cross-sectional	On ART
Duko & Abraham [30]	2018	Ethiopia	395	91.15	IHDS	Low risk	41.1%	Cross-sectional	On ART
Jumare J et al. [31]	2020	Nigeria	190	100	Frascati	Low risk	24.2%	Cross-sectional	On ART
Yitbarek Y et al. [32]	2019	Ethiopia	338	97	IHDS	Low risk	35.7%	Cross-sectional	On ART
Araya et al. [33]	2020	Ethiopia	584	99.5	IHDS	Low risk	35.6%	Cross-sectional	On ART
Yusuf A et al. [34]	2017	Nigeria	418	100	IHDS	Low risk	21.5%	Cross-sectional	On ART
Tareke et al. [35]	2022	Ethiopia	410	96	IHDS	Low risk	66.8%	Cross-sectional	ART naïve patients
Tsegaw et al. [36]	2016	Ethiopia	595	99	IHDS	Low risk	36.4%	Cross-sectional	On ART
Yakasai A et al. [37]	2015	Nigeria	80	100	Frascati	Low risk	35%	Cross-sectional	On ART
Sumonu T et al. [38]	2017	Nigeria	70	72	CSID	Low risk	68%	Cohort	Unknown
Oshinaike et al. [39]	2012	Nigeria	208	100	IHDS	Low risk	54.3%	Case–control	On ART
Salahuddin et al. [40]	2020	Ethiopia	250	96	IHDS	Low risk	39.34%	Cross-sectional	On ART
Milanini et al. [41]	2020	Uganda	500	100	GDS	Low risk	29%	Case–control	Both ART and naïve
Mohamed et al. [42]	2020	Kenya	360	100	IHDS	Low risk	81.1%	Cross-sectional	On ART
Namagga et al. [43]	2019	Uganda	393	100	IHDS	Low risk	58.3%	Cross-sectional	Both ART and naive
Milanini et al. [41]	2020	Tanzania	469	100	GDS	Low risk	52%	Case–control	Both ART and naïve
Mugendi Aet al [16]	2019	Kenya	345	100	IHDS	Low risk	88%	Cross-sectional	On ART
Kelly C et al. [44]	2014	Malawi	106	100	Frascati	Low risk	55%	Cross-sectional	On ART
Milanini et al. [41]	2020	Kenya	1503	100	GDS	Low risk	37%	Case–control	Both ART and naïve
Sacktor N et al. [45]	2019	Uganda	399	87	Frascati	Low risk	51%	Cohort	Both ART and naive
Nyundo A [46]	2022	Tanzania	397	100	MoCA	Low risk	67%	Cross-sectional	On ART
Royal W et al. [47]	2012	Nigeria	60	100	IHDS	Low risk	28.8%	Case–control	On ART
Nyamayaro et al. [48]	2020	Zimbabwe	155	100	GDS	Low risk	49.7%	Case–control	On ART
Fiagbe D et al. [49]	2021	Ghana	123	100	RQCST	Low risk	54%	Cross-sectional	On ART
Asiedu et al. [50]	2020	Ghana	104	100	IHDS	Low risk	48%	Cross-sectional	On ART
Sanmarti et al. [51]	2021	Tanzania	245	99	Frascati	Low risk	19.3%	Cross-sectional	On ART
Joska J et al. [52]	2009	S. Africa	536	100	IHDS	Low risk	23.5%	Cross-sectional	On ART
Joska J et al. [53]	2011	S. Africa	170	100	Frascati	Low risk	76%	Cross-sectional	ART naive patients
Jade C et al. [54]	2017	S. Africa	149	98	IHDS	Low risk	53%	Cross-sectional	ART naive patients
Serfo F et al. [55]	2021	Ghana	500	100	Frascati	Low risk	28.4%	Cohort	Both ART and naïve
Lawler K et al. [26]	2010	Botswana	120	100	IHDS	Low risk	38%	Cross-sectional	On ART
Pascal M et al. [56]	2016	C.Africa R	244	100	IHDS	Low risk	25%	Cross-sectional	On ART
Atashili et al. [25]	2013	Cameroon	400	100	IHDS	Low risk	85%	Cross-sectional	On ART
Awari et al. [57]	2018	Kenya	218	98.6	MoCA	Low risk	89%	Cross-sectional	Both ART and naive
Yechoor et al. [58]	2016	Uganda	181	100	GDS	Low risk	38%	Cross-sectional	On ART
Nakasujja et al. [59]	2012	Uganda	156	100	IHDS	Low risk	64.7%	Cross-sectional	Unknown
Hestad K et al. [60]	2019	Zambia	237	100	GDS	Low risk	34%	Case–control	On ART
Robbins et al. [61]	2011	S. Africa	65	100	IHDS	Low risk	80%	Cross-sectional	ART naive patients
Hulguin et al. [62]	2011	Zambia	54	100	IHDS	Low risk	22%	Case–control	Unknown
Mpungu et al. [63]	2011	Uganda	500	100	IHDS	Low risk	62.8%	Cross-sectional	On ART
Birbeck et al. [64]	2011	Zambia	486	89	IHDS	Low risk	42.1%	Cohort	Both ART and naive
Njamnshi et al. [65]	2009	Cameroon	185	100	IHDS	Low risk	22.2%	Cross-sectional	On ART
Patel V et al. [15]	2010	Malawi	179	100	IHDS	Low risk	14%	Cross-sectional	Both ART and naive
Kalungwana et al. [66]	2014	Zambia	58	100	IHDS	Low risk	53%	Cross-sectional	Unknown
Sacktor N et al. [67]	2009	Uganda	60	100	IDHS	Low risk	35%	Cross-sectional	ART naive patients
Sacktor N et al. [68]	2009	Uganda	102	100	IDHS	Low risk	39.2%	Cohort	ART naive patients

*ART* Anti-Retroviral Therapy, *GDS* Global Deficit Score, *IHDS* International HIV Dementia scale, *MoCA* Montreal Cognitive Assessment, *HIV* Human Immunodeficiency Virus

### Prevalence of neurocognitive impairment

**Fig. 2:**
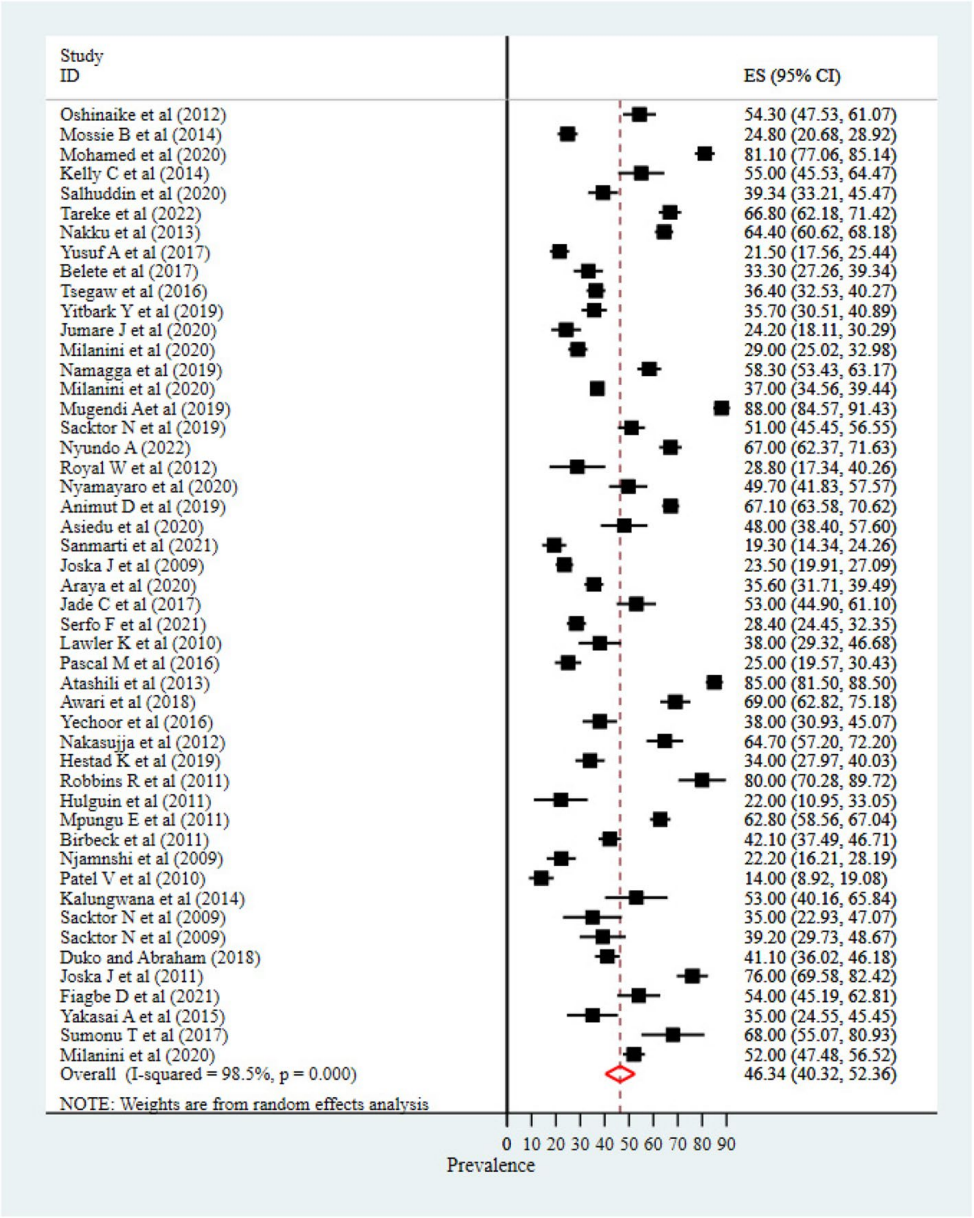
Pooled prevalence of HIV/AIDS associated neurocognitive impairment in Africa

The prevalence of HIV/AIDS-associated neurocognitive impairments was highly variable across studies, ranging from 14% to 88%, and I^2^ = 98.5%, yielding the pooled prevalence of HIV/AIDS-associated neurocognitive impairments to be 46.34% [95% CI (40.32, 52.36)] (Fig. 2).

### Sub-group analysis results

The subgroup analysis of HIV/AIDS-associated neurocognitive impairments showed a reduction in the percentage of heterogeneity in terms of geographical locations of studies conducted, study design, measurement tools, and participants’ ART status. The highest I^2^ drop was observed when investigating a subgroup analysis using measurement tools.

### Difference in screening tools

**Fig. 3:**
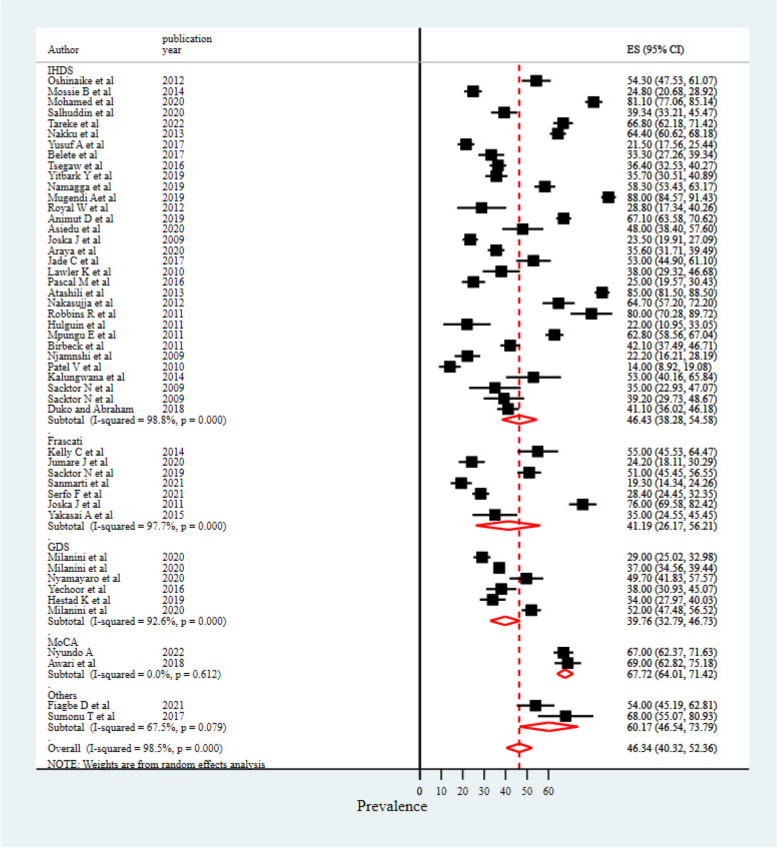
Subgroup analysis result based on screening tools

For measurement tools, the highest prevalence (67.72%) of neurocognitive impairments was observed among studies that used the "Montreal cognitive assessment (MoCA)" to measure neurocognitive impairments, followed by studies that used the "international HIV dementia scale" (46.43%). However, only two studies used MoCA screening tools to measure HIV/AIDS-associated cognitive disorder, which may make it difficult to compare the average percentage of these two studies with others. The lowest prevalence was reported from studies that used the "global deficit score" in measuring the level of HIV/AIDS-associated cognitive impairments (Fig. 3).

### Differences in study designs

**Fig. 4:**
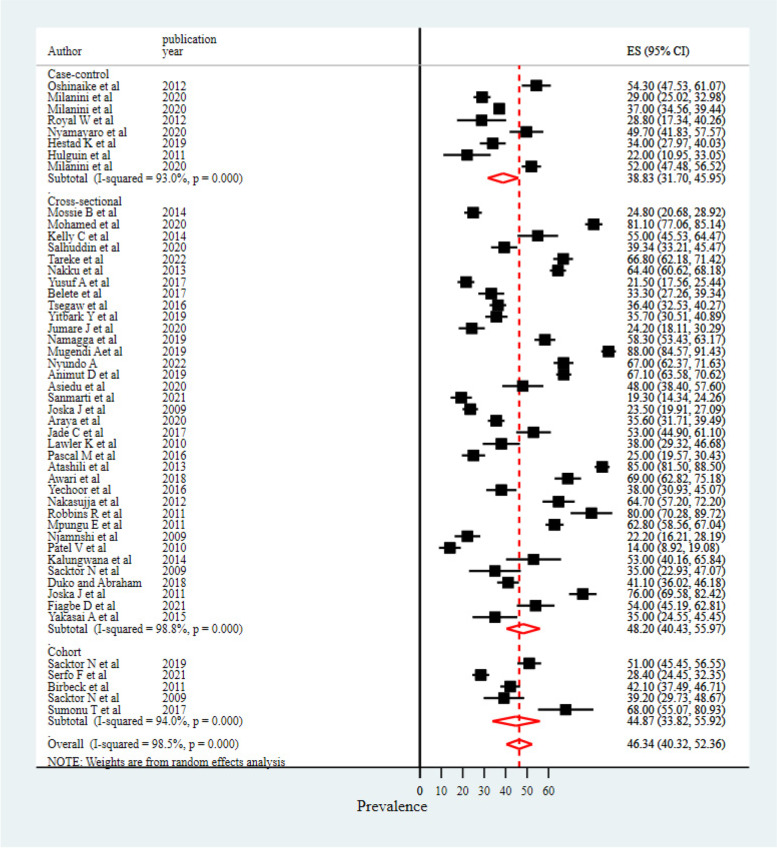
Subgroup analysis result based on study deign

We included observational studies (cross-sectional, case–control, and cohort studies) conducted across 15 African countries. Significant variability in reported results was observed across studies based on differences in their study designs. Cross-sectional studies showed the highest rate of neurocognitive impairments as compared to other study designs (48.20%) (Fig. 4).

### Differences in geographical locations

**Fig. 5:**
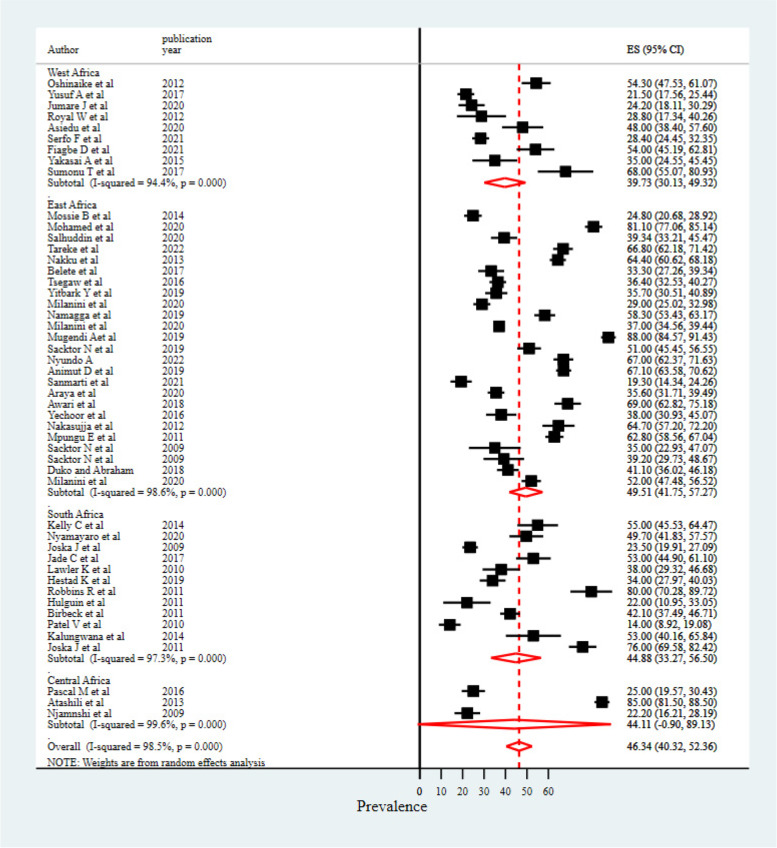
Subgroup analysis result based on study locations

Studies from the eastern regions of Africa showed the highest prevalence of neurocognitive impairments (49.51%), and the lowest prevalence was reported for studies from the western regions of the continent (39.73%). However, the prevalence of neurocognitive impairment among people living with HIV/AIDS in the studies carried out in the southern and central regions remained relatively similar (44.88% and 44.11%, respectively) (Fig. 5).

### Differences in ART status

**Fig. 6:**
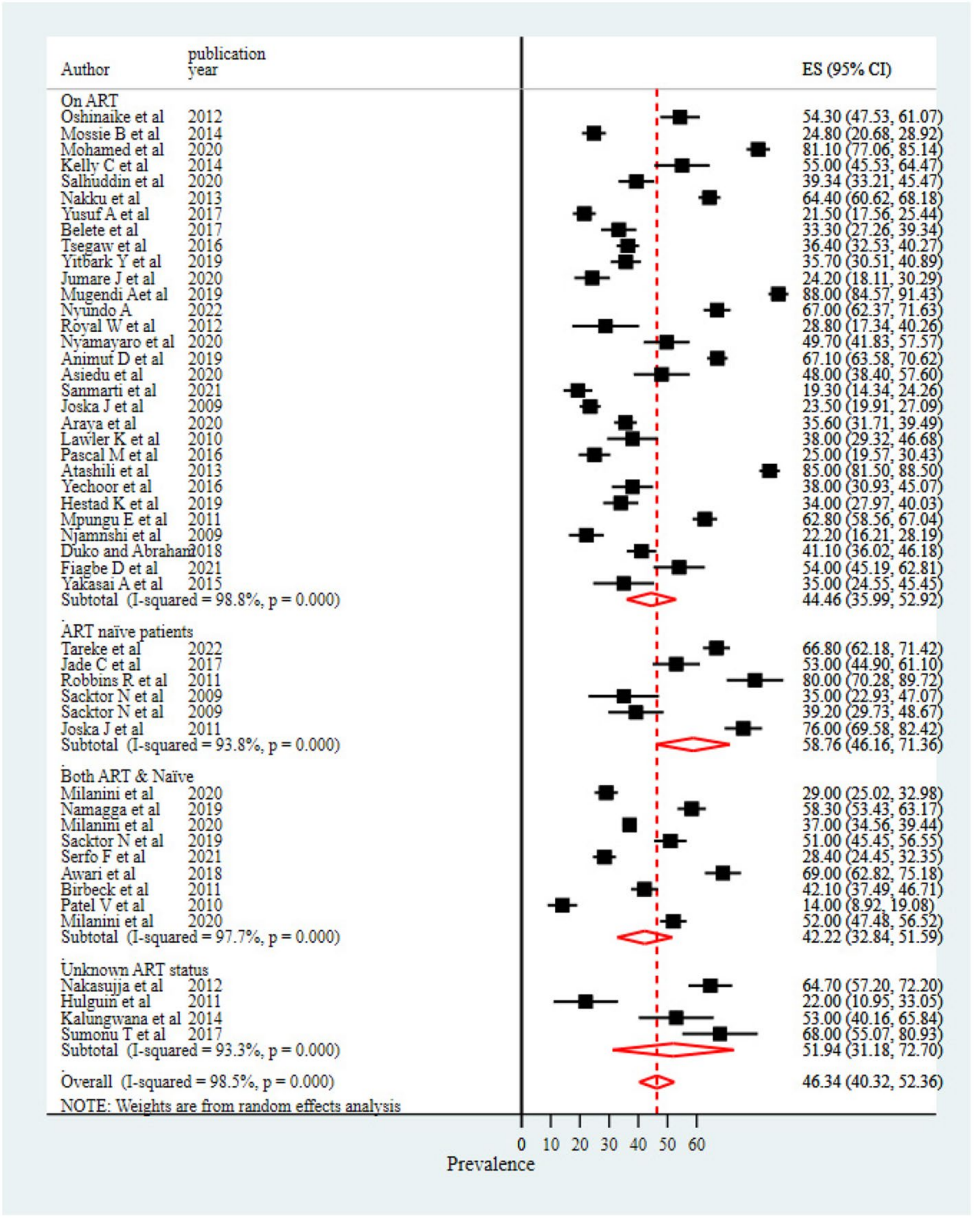
Subgroup analysis result by ART status of enrolled participants

The subgroup analysis result based on the ART status of the study participants revealed that patients with no ART experience showed a higher prevalence of cognitive impairments (58.76%), followed by study participants with unknown ART status (51.94%) (Fig. 6).

### Publication bias

**Fig. 7:**
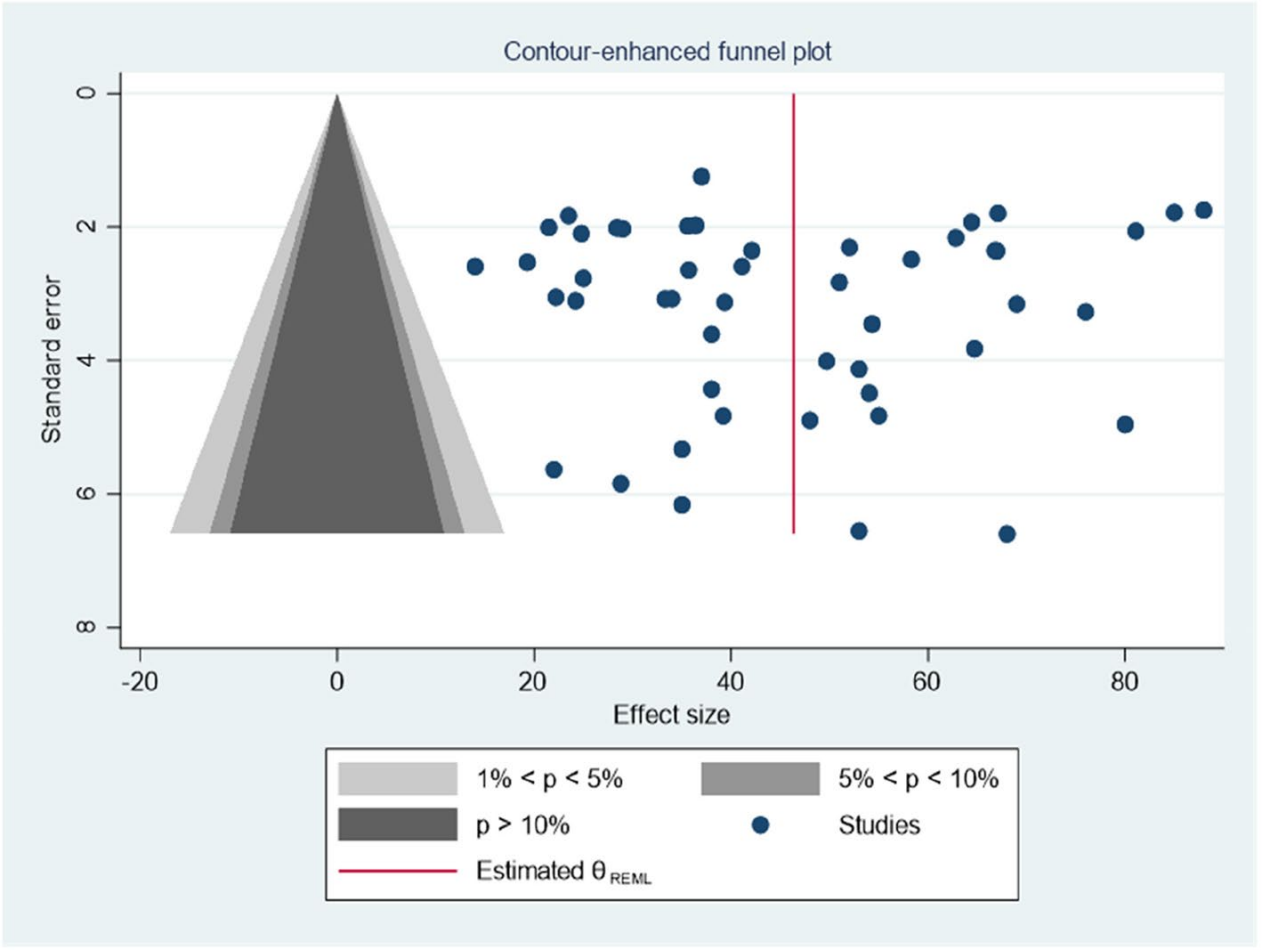
Counter enhanced funnel plot

The graphical inspection indicated even distributions of estimates of each study around the mean effect size, with no imputed study in the contour-enhanced funnel plot for a logit event rate of occurrence of HIV-associated neurocognitive impairmets in people living with HIV/AIDS, alongside its standard error, suggesting evidence of a symmetrical distribution. In addition, the quantitative Eggers test results of publication bias, *P* = 0.71 (non-significant) suggested that there was no publication bias in our systematic review and meta-analysis (Fig. 7).

### Meta regression

**Fig. 8:**
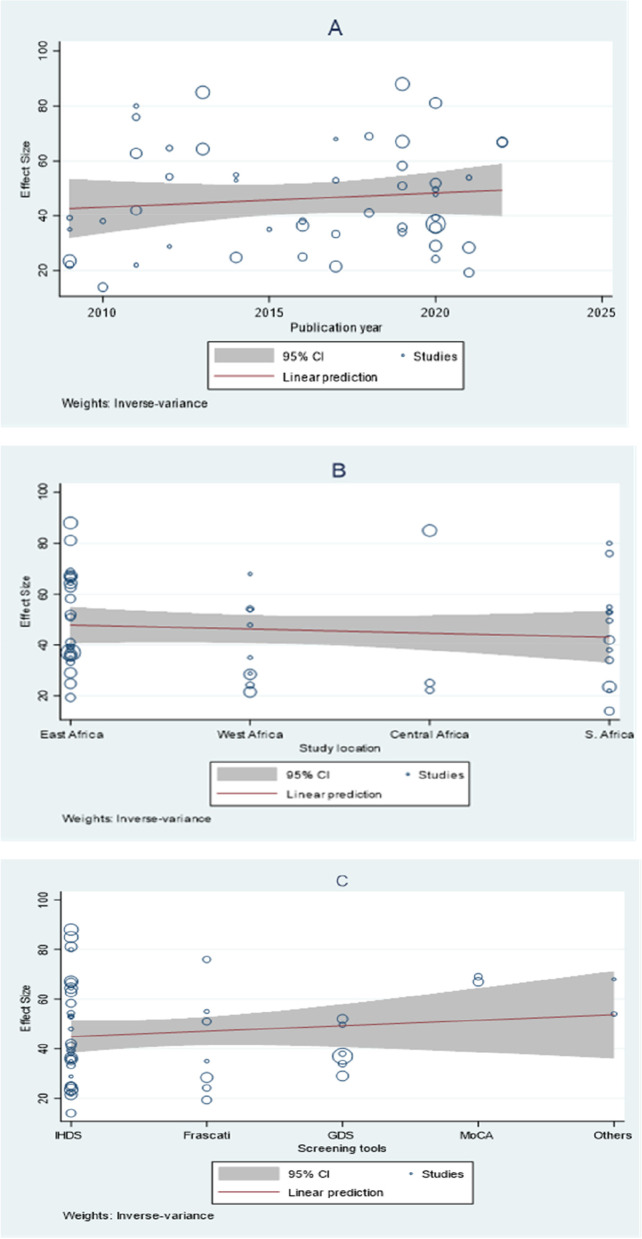
Meta regression result (A = publication year, B = study location and C = screening tools)

A meta-regression has been conducted based on publication years, screening tools, and the geographical location of primary studies. The trend of the meta-regression result showed that there was no significant change over the linear prediction of studies in all parameters (publication year, study location, and screening tools for neurocognitive impairments) (Fig. 8).

## Discussion

Despite the reduction in morbidity and mortality among people with HIV/AIDS after the introduction of ART, neurocognitive impairments remain a public health concern due to the chronic nature of HIV/AIDS. The problem becames a serious issue, particularly in sub-Saharan countries as a result of late initiation of ART, opportunistic infections, lower awareness of HIV/AIDS treatments, frequent nonadherence to anti-retroviral therapy (ART), and the poor health care systems in the region [69]. This negatively affects the patients' quality of life, functionality, treatment adherence, health care costs, health care workloads, viral load suppression, and the overall survival rate of people with HIV/AIDS [7, 70].

The result of this systematic review revealed that nearly half (46.36%) of people living with HIV/AIDS in Africa have some level of neurocognitive impairments (asymptomatic neurocognitive impairment, mild neurocognitive disorder, or HIV-associated dementia). This figure is higher than the prevalence of neurocognitive impairments reported by other studies conducted at the international level (20–37%) published in 2009 [71], a study from sub-Saharan settings (30–39%) published in 2013 [72], a global level study (42,6%) published in 2020 [14] and another similar systematic review (44.46%) which is published in 2020 [73].

The higher prevalence of neurocognitive impairments in this systematic review might be explained by inadequate health care coverage in Africa. Moreover, the newly innovated positive mental health and recovery approach have not been properly implemented, and the healthcare model in Africa merely focused on the traditional pharmacological treatment approaches. Thus, professionals are currently recommended to integrate psychosocial support services as an added treatment modality for people with HIV/AIDS to reduce the development of cognitive problems. On the other hand, the pooled prevalence of neurocognitive impairments in this systematic review was lower than the findings of another similar study (50.6%) [74]. The social cohesion and collective culture of African communities might play roles in delivering informal psychosocial support services. In addition, the extended family structure in Africa potentialy increases the opportunity for individuals to have more social connections which enhance their cognitive performance.

The data regarding the prevalence of neurocognitive impairments reported across studies was highly heterogeneous, making it hard to compare the magnitude and evaluate the impacts of intervention programs for people with HIV/AIDS, as well as to analyse trends of neurocognitive impairments over time.

The percentage of data variability has been reduced when we clustered studies with differences in the screening tools, study designs, geographical locations, and participants’ ART status. Accordingly, people with ART status showed the lowest I^2^ drop in the prevalence of neurocognitive impairments as compared with other parameters of variability assessment. Regarding study design, case control studies had the highest I^2^ drop.

On the other hand, studies that used MoCA and IDHS tools to measure the prevalence of neurocognitive impairments showed increased prevalences (67% and 46.4%, respectively). However, the lowest prevalence was reported among studies that used GDS to measure neurocognitive impairments (39.7%). The highest prevalence of neurocognitive impairments among studies that used MoCA and IDHS screening tools is potentially explained by the screening nature of the tools (higher sensitivity), which cannot confirm the clinical diagnosis of neurocognitive impairment [61, 75] as compared to the global deficit score, which is relatively comprehensive and has multiple cognitive tests that includes the severity of impairment [76]. This calls for practical approaches to codesign and establish valid and standardized neurocognitive impairment measurement tools, particularly applicable to the African context. Cross-sectional studies showed the highest magnitude of neurocognitive impairments as compared to studies with other designs. This might be due to the inability of cross-sectional study designs to determine other confounders, and the prevalence of neurocognitive impairments might be overestimated among people with HIV/AIDS. The difference in terms of study locations revealed that studies from the eastern region of Africa showed the highest prevalence of neurocognitive impairments (49.5%), while the lowest prevalence rate was reported among studies conducted in the western region of the African population. In addition, the subgroup analysis results computed by ART status of enrolled participants showed that people with no ART experience were more likely to develop cognitive impairments (56.76%). This indicates that ART drugs with proper management have significant impacts in reducing or preventing cognitive problems [77].

### Limitation

This systematic review has three limitations. 1/The heterogeneous nature of the data on the pooled prevalence of HIV/AIDS-associated neurocognitive impairments might reduce its validity to draw conclusions. 2/ Measurement tools of neuro-cognitive impairments used by all included studies were screening tools (they did not confirm a diagnosis), and the figure for the actual HIV/AIDS-associated neuro-cognitive disorder has never been confirmed. 3/ Other factors (e.g., substance and psychosocial problems) that might affect the prevalence of HIV/AIDS-associated neuro-cognitive impairments have not been investigated in this review.

## Conclusions

A significant proportion of people living with HIV/AIDS in Africa have associated neurocognitive impairments. Data regarding HIV/AIDS-associated neurocognitive impairments was highly variable across studies and partially explained by differences in the assessment tools/batteries used. Designing and establishing standardized screening tools validated for the African context is highly recommended to make comparisons and draw conclusions on the prevalence of HIV/AIDS-associated neurocognitive impairments.

## Supplementary Information

**Supplementary file 1.**

**Supplementary file 2.**

## Data Availability

All data included in this manuscript are available and can be accessed by the corresponding author with a reasonable request.

## Abbreviations

AIDS: Acquired Immune Deficiency Syndrome
ART: Antiretroviral Therapy, HAND: HIV-Associated Neurocognitive Disorder
CD4: Cell Differentiation
CI: Confidence Interval
IHDS: International HIV Dementia Scale
PRISMA: Preferred Reporting Items for Systematic reviews and Meta-Analyses
