# Supplementary material for: Prevalence and variability of HIV/AIDS-associated neurocognitive impairments in Africa: a systematic review and meta-analysis
Source: BMC Public Health. 2023 May 30;23:997. doi: 10.1186/s12889-023-15935-x (PMC10228136; doi:10.1186/s12889-023-15935-x)
Supplement: Supplementary file 2 [file 12889_2023_15935_MOESM2_ESM.docx]

Revised PICO (Population, Intervention, concept/context, and Outcome) model of our search strategies

| **P (Population)** | **I (intervention)** | **C (context/concept)** | **O(outcome)** |
| --- | --- | --- | --- |
| "People with HIV/AIDS*"  "sero?positive*"  "HIV/AIDS population*"  "ART Outpatient*"  "HIV/AIDS patient*"  "HIV positive"  "ART-naive patient*" | "Cognitive impairment*"  "Cognitive problem*"  "Cognitive functioning*"  "Neuro?cognitive impairment*"  "AIDS dementia*"  " Neuro?cognitive disorder*"  " Neuro?cognitive disease*"  "Cognitive level*" | "ART clinic*"  "HAART clinic*"  "HIV/AIDS "  "ART follow?up *"  "HIV testing and counselling"  "Professional Initiated counselling and testing"  "Chronic care*"  "HIV/AIDS service*" | "prevalence*"  "magnitude*"  "epidemiology*"  "burden*"  "frequency*"  "rate*"  "level*"  "incidence*"  "Risk factor*" |

**P-** ("People with HIV/AIDS*" OR "ART-naive patient*" OR "sero?positive*" OR "HIV/AIDS population*" OR "ART Outpatient*" OR "HIV/AIDS patient*" OR "HIV positive")

**I-** ("Cognitive impairment*" OR "Cognitive problem*" OR "Cognitive functioning*" OR "Neuro-cognitive impairment*" OR "AIDS dementia*" OR " Neurocognitive disorder*" OR "Neuro cognitive disease*" OR "Cognitive level*")

**C-** ("ART clinic*" OR "HAART clinic*" OR "HIV/AIDS " OR "ART follow?up *" OR "HIV testing and counseling" OR "Professional Initiated counseling and testing" OR "HIV/AIDS service*" OR "Chronic care*")

**O-** ("prevalence*" OR "magnitude*" OR "epidemiology*" OR "burden*" OR "frequency*" OR "rate*" OR "level*" OR "incidence*" OR "risk factor*")
